# Supplementary figures and images for: Novel tools for comparing the architecture of psychopathology between neurogenetic disorders: An application to X- versus Y-chromosome aneuploidy effects in males
Source: Psychol Med. 2025 Jun 17;55:e166. doi: 10.1017/S0033291725000765 (PMC12180508; doi:10.1017/S0033291725000765)

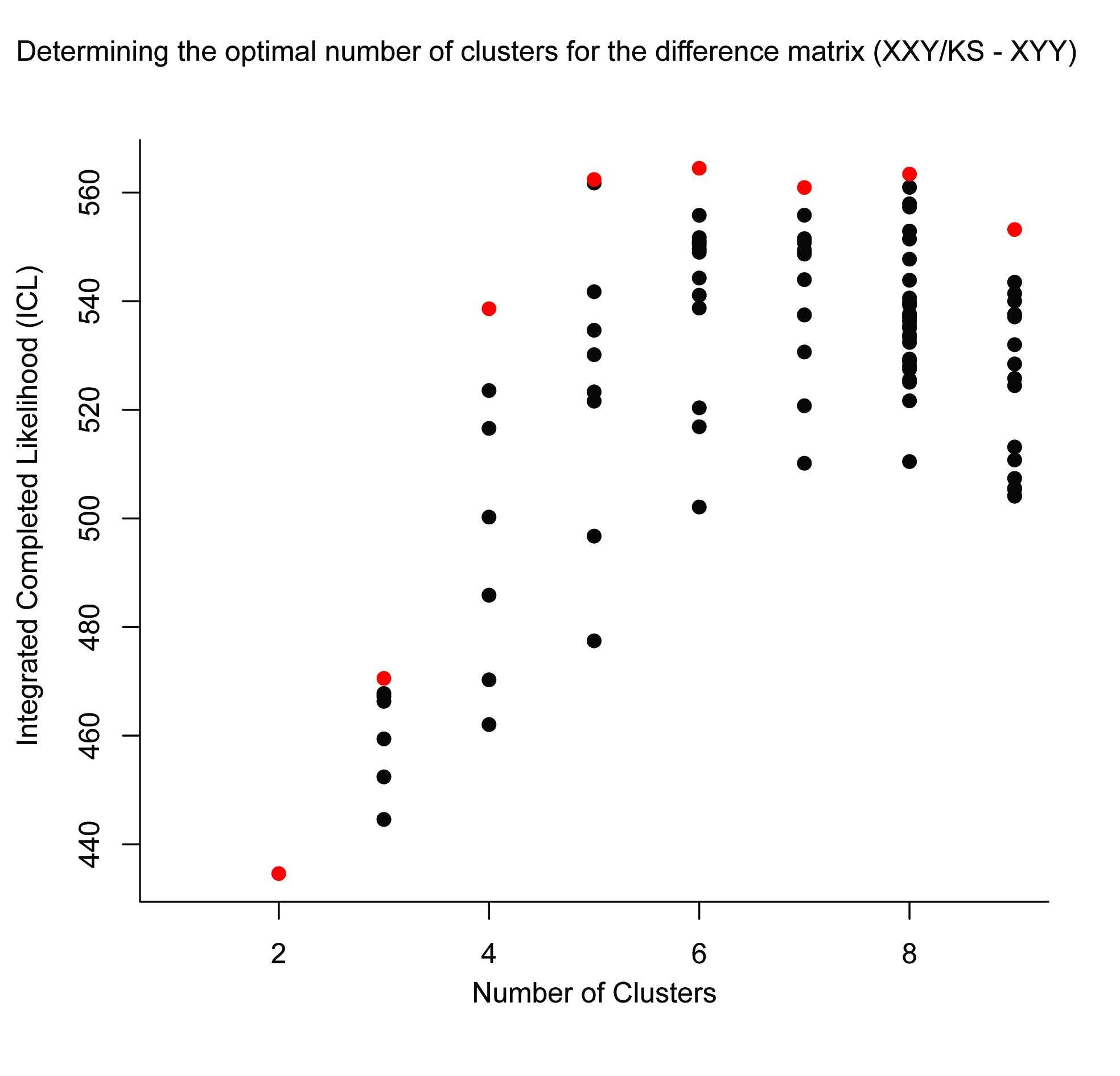

Supplement: Larsen et al. supplementary material 1 — Larsen et al. supplementary material [file S0033291725000765sup001.tif]
